# Real-Time qPCR Identifies Suitable Reference Genes for Borna Disease Virus-Infected Rat Cortical Neurons

**DOI:** 10.3390/ijms151221825

**Published:** 2014-11-26

**Authors:** Lujun Zhang, Siwen Liu, Liang Zhang, Hongmin You, Rongzhong Huang, Lin Sun, Peng He, Shigang Chen, Hong Zhang, Peng Xie

**Affiliations:** 1 Department of Neurology, Yongchuan Hospital, Chongqing Medical University, Chongqing 402460, China; E-Mail: chongqing2012bruce@gmail.com; 2 Chongqing Key Laboratory of Neurobiology, Chongqing 400016, China; E-Mails: 18983145528@163.com (S.L.); zhlbright@gmail.com (L.Z.); yhm0709@126.com (H.Y.); tilamisu789456123@126.com (L.S.); hepeng000@sina.com (P.H.); iconsig@sina.com (S.C); ASDFG43215@126.com (H.Z.); 3 Institute of Neuroscience, Chongqing Medical University, Chongqing 400016, China; 4 Department of Neurology, the First Affiliated Hospital of Chongqing Medical University, Chongqing 400016, China; 5 Department of Rehabilitation, the Second Affiliated Hospital, Chongqing Medical University, Chongqing 400016, China; E-Mail: rzh uang@live.com

**Keywords:** Borna disease virus, BDV, reference gene, RT-qPCR, cortical neuron

## Abstract

Quantitative real-time reverse transcription polymerase chain reaction (RT-qPCR) is the most commonly-used technique to identify gene expression profiles. The selection of stably expressed reference genes is a prerequisite to properly evaluating gene expression. Here, the suitability of commonly-used reference genes in normalizing RT-qPCR assays of mRNA expression in cultured rat cortical neurons infected with Borna disease virus (BDV) was assessed. The expressions of eight commonly-used reference genes were comparatively analyzed in BDV-infected rat cortical neurons and non-infected control neurons mainly across 9 and 12 days post-infection. These reference genes were validated by RT-qPCR and separately ranked by four statistical algorithms: geNorm, NormFinder, BestKeeper and the comparative delta-*C*t method. Then, the RankAggreg package was used to construct consensus rankings. *ARBP* was found to be the most stable internal control gene at Day 9, and *ACTB* at Day 12. As the assessment of the validity of the selected reference genes confirms the suitability of applying a combination of the two most stable references genes, combining the two most stable genes for normalization of RT-qPCR studies in BDV-infected rat cortical neurons is recommended at each time point. This study can contribute to improving BDV research by providing the means by which to obtain more reliable and accurate gene expression measurements.

## 1. Introduction

Borna disease virus (BDV) is the causative agent of Borna disease, an enzootic encephalomyelitis of horses and sheep named after epidemics having occurred in horses close to the city of Borna in Saxony (Germany) at the end of the 19th century. BDV is a neurotropic, non-cytolytic, non-segmented, negative-stranded RNA virus belonging to the order, Mononegavirales. The BDV genome spans approximately 8.9 k band consists of six major open reading frames (ORFs). It is a neurotropic RNA virus that can infect many vertebrate species [1], including man. Whether or not BDV is involved in human disease, like mental disorders, remains a controversial issue [2]. To date, BDV infection has been reported in a range of animal species across a broad global geographic distribution [3], including China [4,5]. Infected hosts develop a wide spectrum of neurological disorders, ranging from immune-mediated diseases to behavioral alterations without inflammation [6]. In rats, neonatal BDV infection causes disturbances in learning, mood and behavior reminiscent of those observed in human psychiatric diseases, which is a model system to study the consequences of persistent viral infections for brain function, morphology and behavior [7].

The mechanism(s) underlying BDV pathogenesis are not well understood [8]. When attempting to analyze the biomolecular consequences of a BDV infection model, real-time quantitative reverse transcription polymerase chain reaction (RT-qPCR) is a well-established, facile technique, because it allows fast, accurate and sensitive evaluation of mRNA levels in biological samples [9]. Proper use of this method requires normalization to account for the differences in the amount of starting material, variability in RNA quality, variable PCR or cDNA synthesis efficiencies and differences between tissues and cell types in overall transcriptional activity. The most frequently applied approach for normalization is the use of reference genes. Thus, normalizing reference genes is a simple and popular method for an internal control of errors in RT-qPCR. Several studies, however, have demonstrated that the expression levels of reference genes can vary under different experimental conditions [10].

To date, no formal evaluation of optimal mRNA reference genes in BDV research has been made. Thus, the aim of this study was to identify the most stable one or a combination of the most stable ones, in cultured primary rat cortical neurons infected with a human BDV. We selected a total of 10 frequently used reference genes (*HPRT*, *YWHAZ*, *TPB*, *Rpl13A*, *GAPDH*, *ACTB*, *PPIA*, *ARBP*, *18sRNA*, *B2M*) as candidate reference genes. They were afterwards validated by RT-qPCR of control neurons and infected ones. Four statistical algorithms (geNorm, NormFinder, BestKeeperand the comparative delta-*C*t method), as well as consensus rankings were applied to identify the most stable reference genes. Thus, our results can provide information about appropriate reference genes for normalization of qPCR data in Sprague-Dawley rat cortical neurons infected with BDV.

## 2. Results

### 2.1. Immunofluorescence Assay

**Figure 1:**
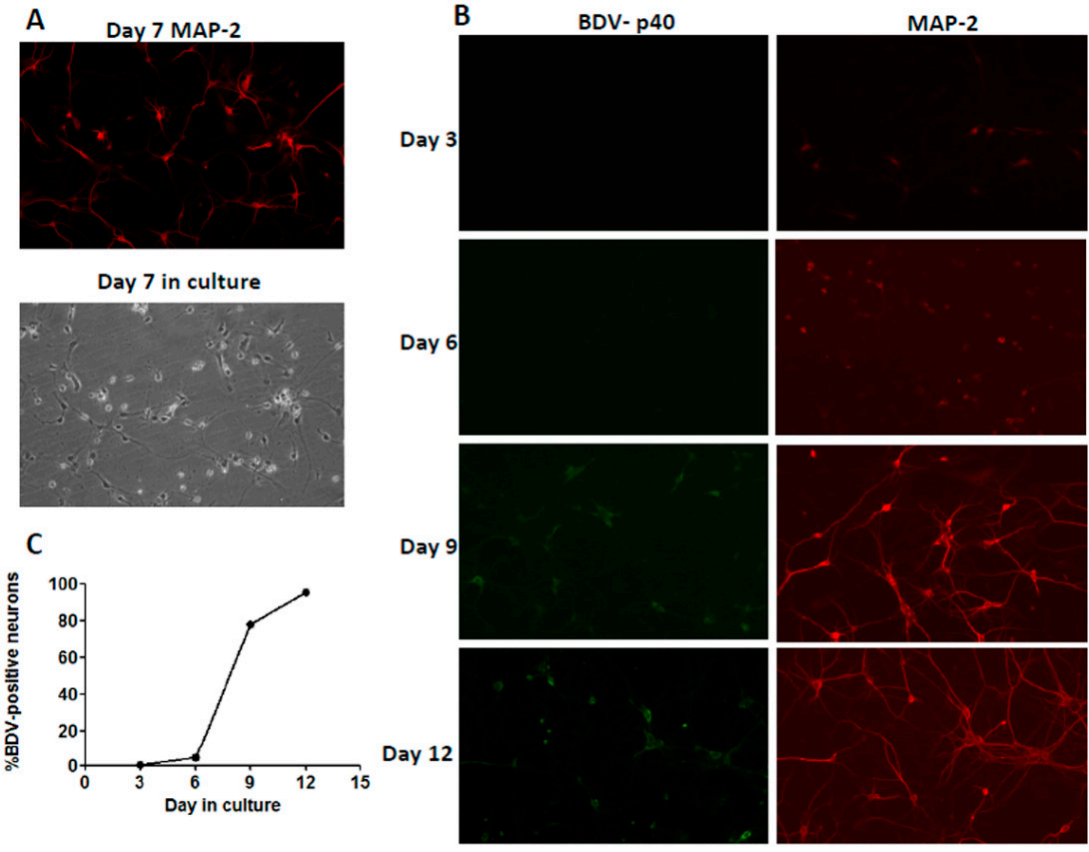
Immunofluorescence analysis of neurons infected with Borna disease virus (BDV). (**A**) Purity of neurons on Day 7; (**B**) BDV P40 (Genscript, Nanjing, China) detected with a primary monoclonal antibody followed by a FITC-labeled secondary antibody (green, Goat anti Mouse) (Abcam, Cambridge, MA, USA). Neurons marked with chicken polyclonal MAP-2 (Abcam) followed by an Alexa Fluor 594-conjugated secondary antibody (red, goat anti chicken) (Abcam) at Days 3, 6, 9 and 12; and (**C**) Quantification of virus spread. On different days of the tissue culture, the percentages of BDVN-positive neurons were assessed.

This test was applied to detect the purity of neurons at day seven post-infection. The percentage of neurons was determined through observation of randomly selected cells across three independent experiments. The results showing the purity of neurons was more than 80% (Figure 1A). Percentages of BDV P40-positive neurons were assessed on Days 3, 6, 9, 12 post-infection. On Days 3, 6, 9, 12 samples were collected and analyzed by immunofluorescence microscopy, and the percentage of BDV P40-positive neurons was quantified. BDV infection was first detectable on Day 6 and BDV P40-positive neurons were less than 4%. Between Days 6 and 9, BDV spread rapidly, and by Day 12, almost 100% of the cells were infected (Figure 1B,C).

### 2.2. Evaluation of Expression Stability

Ten candidate reference genes were chosen from the literature. Their full names, primer sequences, amplicon lengths and amplification efficiencies are shown in Table 1. We used quantitative real-time RT-PCR to evaluate the expression of these candidate reference genes in rat cortical neurons between the BDV infected group *versus* (*vs*.) control group *in vitro* at 9 days and 12 days post-infection. All candidate reference gene products revealed single bands for all primer sets during agarose gel electrophoresis (*not shown*). Melting curve analysis consistently demonstrated a single homogenous melting peak for each primer set. Moreover, no amplicons were detected for the NTC (no template control) cells. The standard curve revealed the amplification efficiencies for all candidate reference genes (Table 1). These results indicated that the method of measurement was appropriate. To identify candidate reference mRNAs from each group, the following criteria were used: (1) RNA normalized gene expression; (2) the fold-change of the candidate reference gene expression between the two groups at two time point was not more than 1.1×; and (3) no significant differences (*p*-value < 0.05) existed between the two groups (using the Student’s *t*-test and the Wilcoxon–Mann–Whitney test). In this study, all candidate reference genes met these criteria, except B2M and 18s rRNA, as there were significant differences for these two candidate reference genes between the two groups, measured at two time points.

### 2.3. Candidate Reference Gene Ranking

**Figure 2:**
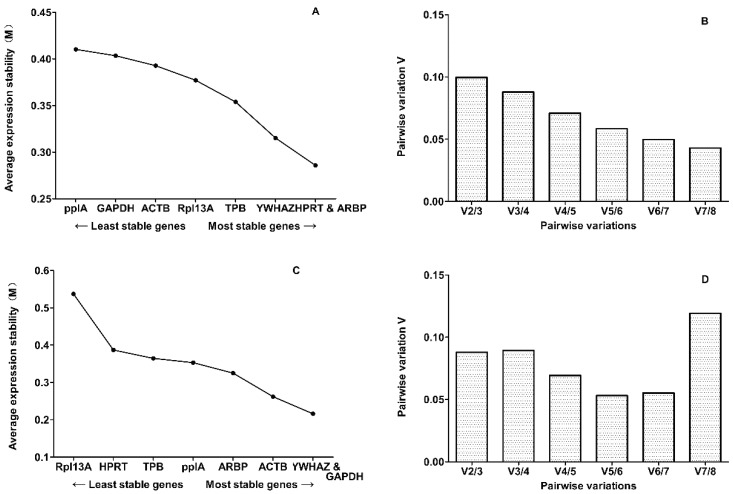
The average expression stability values (*M*) calculated by geNorm across the two points. Average expression stability measure (*M*) of eight reference genes at (**A**) Day 9, (**C**) Day 12, respectively, during stepwise exclusion of the least stable control gene. A lower *M*-value of the average expression stability indicates a more stable expression. Determination of the optimal number of control genes for normalization on the basis of a pair-wise variation (Vn/n + 1) analysis. Every bar represents a change in normalization accuracy when stepwise adding more endogenous controls according to ranking at (**B**) Day 9, (**D**) Day 12.

Only eight candidate reference genes (HPRT, YWHAZ, TPB, Rpl13A, GAPDH, ACTB, PPIA and ARBP) met the criteria and were subsequently tested for the stability of gene expression with the four aforementioned algorithms. GeNorm [11] was used to rank the candidate reference genes on the basis of their expression stability value (M) to identify the most stable reference gene at each of the four time points. The lowest M-value corresponds to the most stable reference gene, while the highest corresponds to the least stable one. In previous studies, an M-value of 1.5 was set as a cut-off to assess gene stability [12]. As shown in Figure 2, geNorm identified ARBP and HPRT as the most stable pair-wise combination of reference genes for the experimental groups at Day 9 (M-value for the combination of the two genes: 0.2859) and GAPDH and YWHAZ at Day 12 (M-value for the combination of the two genes: 0.2159). Moreover, geNorm calculates a normalization factor (*V/NF value*) that is a criterion for the optimum number of reference genes (Figure 2). According to Vandesompele et al. [11], the ideal pair-wise variation value is less than 0.15. In our data sets, the calculated V2/3 was less than 0.15. Thus, there is no need to include more than two genes into the normalization factor, and only the two most stable genes are needed for a reliable normalization.

NormFinder [13] uses a model-based approach to estimate overall reference gene stability, but also considers variations between sample subgroups. It enables the identification of the single best gene, as well as providing a ranking order. NormFinder identified *ARBP* and *ACTB* as the best two reference genes for Day 9and *ACTB* and *YWHAZ* as the best ones for Day 12 (Table 2).

**Table 1:** Primers used for real-time PCR.

Symbol	Accession	Name	Forward and Reverse Primer (5'-3')	Amplicon Size	Primer Efficiency	Cq Value (Average ± SD)	Reference ^a^	Mean Relative Quantification ^b^(9, 12 Days)
*HPRT*	NM012583	Hypoxanthine phosphoribosyl-transferase	CTCATGGACTGATTATGGACAGGACGCAGGTCAGCAAAGAACTTATAGCC	123	93	23.36 ± 0.78	[10]	0.83; 1.16
*YWHAZ*	NM013011	Tyrosine 3-monooxygenase/tryptophan 5-monooxygenase activation protein, zeta polypeptide	GATGAAGCCATTGCTGAACTTGGTCTCCTTGGGTATCCGATGTC	117	100	19.04 ± 0.66	[10]	0.78; 0.91
*TPB*	NM001004198	TATA box binding protein	TGGGATTGTACCACAGCTCCACTCATGATGACTGCAGCAAACC	131	93	23.94 ± 0.65	[10]	0.91; 1.25
*Rpl13A*	NM173340	Ribosomal protein L13A	GGATCCCTCCACCCTATGACACTGGTACTTCCACCCGACCTC	132	94	18.67 ± 1.12	[10]	0.99; 1.28
*GAPDH*	NM017008	Glyceraldehyde-3-phosphate dehydrogenase	CAACTCCCTCAAGATTGTCAGCAAGGCATGGACTGTGGTCATGA	118	105	20.35 ± 0.67	[14]	0.80; 0.78
*ACTB*	NM031144	Beta-actin	CAGGGTGTGATGGTGGGTATGGAGTTGGTGACAATGCCGTGTTC	115	103	17.88 ± 0.85	[15]	1.00; 0.99
*PPIA*	NM017101	peptidylprolylisomerase A	GTCAACCCCACCGTGTTCTTCATCCTTTCTCCCCAGTGCTCAG	133	93	17.06 ± 0.82	[15]	0.85; 0.97
*ARBP*	NM022402	Acidic ribosomal phosphoprotein P0	TAGAGGGTGTCCGCAATGTGCAGTGGGAAGGTGTAGTCAGTC	137	102	22.02 ± 0.62	[16]	0.81; 0.73
*B2M*	NM012512	Beta-2 microglobulin	CGAGACCGATGTATATGCTTGCGTCCAGATGATTCAGAGCTCCA	114	92	18.16 ± 1.15	[14]	2.51; 3.24
*18s rRNA*	M11188	18s subunit ribosomal RNA	ACGGACCAGAGCGAAAGCATTGTCAATCCTGTCCGTGTCC	310	107	18.07±0.76	[17]	0.72; 0.69
*BDNF*	NM001270630	Brain-derived neurotrophic factor	ACAGTATTAGCGAGTGGGATTGGGTAGTTCGGCATT	213	96	25.55 ± 0.30	a	-
*AldoC*	NM012497	Aldolase C, fructose bisphosphate	ACCTGGAAGGGACTCTCCTCAAAGTCACCCCTGGGACAGCT	141	103	20.86 ± 0.77	[18]	-

^a^ Primers were designed by our laboratory using Primer Premier 5 software; ^b^ mean fold change of each gene mRNA level (infected *vs*. non-infected) at 9 and 12 days; fold change = 2^−[(Mean Cq) infected group − (Mean Cq) non-infected group]^.

**Table 2:** Ranking of reference genes by geNorm, NormFinder, BestKeeper and delta-*C*t. Reference genes are ranked in two groups at each time point as follows: C + V = control group and infected group, *n* = number of independent determinations. Genes are ranked by their stability values calculated by the outputs of the four programs: geNorm, *M*-value; NormFinder, variability; BestKeeper, coefficient of correlation (*r*); delta-*C*t, mean standard deviation.

Genorm	9 Days (C + V, *n* = 12)		12 Days (C + V, *n* = 12)	
**Rank**	**Gene**	***M*-Value**	**Gene**	***M*-Value**
1	*ARBP*	0.2859	*GAPDH*	0.2159
2	*HPRT*	0.2859	*YWHAZ*	0.2159
3	*YWHAZ*	0.3152	*ACTB*	0.2617
4	*TPB*	0.3539	*ARBP*	0.3249
5	*Rpl13A*	0.3771	*ppIA*	0.3529
6	*ACTB*	0.3928	*TPB*	0.3645
7	*GAPDH*	0.4035	*HPRT*	0.387
8	*ppIA*	0.4103	*Rpl13A*	0.537
**NormFinder**	**9 Days (C + V, *n* = 12)**		**12 Days (C + V, *n* = 12)**	
**Rank**	**Gene**	**Variability**	**Gene**	**Variability**
1	*ARBP*	0.0565	*ACTB*	0.0709
2	*ACTB*	0.0627	*YWHAZ*	0.1131
3	*TPB*	0.0713	*ppIA*	0.1363
4	*YWHAZ*	0.0796	*TPB*	0.1521
5	*HPRT*	0.0815	*HPRT*	0.1626
6	*Rpl13A*	0.0821	*GAPDH*	0.1643
7	*GAPDH*	0.0873	*ARBP*	0.199
8	*ppIA*	0.1055	*Rpl13A*	0.3008
**BestKeeper**	**9 Days (C + V, *n* = 12)**		**12 Days (C + V, *n* = 12)**	
**Rank**	**Gene**	**(*r*)**	**Gene**	**(*r*)**
1	*ppIA*	0.879	*ppIA*	0.874
2	*ARBP*	0.868	*TPB*	0.809
3	*GAPDH*	0.784	*ACTB*	0.749
4	*YWHAZ*	0.72	*ARBP*	0.734
5	*HPRT*	0.703	*HPRT*	0.584
6	*ACTB*	0.7	*YWHAZ*	0.583
7	*Rpl13A*	0.527	*GAPDH*	0.511
8	*TPB*	0.497	*Rpl13A*	0.146
**Delta-*C*t**	**9 Days (C + V, *n* = 12)**		**12 Days (C + V, *n* = 12)**	
**Rank**	**Gene**	**Mean SD**	**Gene**	**Mean SD**
1	*ARBP*	0.34	*ACTB*	0.42
2	*TPB*	0.4	*YWHAZ*	0.45
3	*GAPDH*	0.4	*TPB*	0.45
4	*HPRT*	0.41	*GAPDH*	0.46
5	*ACTB*	0.41	*ARBP*	0.49
6	*Rpl13A*	0.42	*ppIA*	0.5
7	*ppIA*	0.42	*HPRT*	0.52
8	*YWHAZ*	0.43	*Rpl13A*	1

BestKeeper [19] uses repeated pair-wise correlation analysis of candidate gene quantification cycle (Cq) values to determine the optimal reference genes. BestKeeper calculates the correlation between the genes and with the BestKeeper index (Pearson correlation coefficient, *r*) [20]. With BestKeeper, *ppIA* and *ARBP* were ranked as the two most stable genes with Cq correlation coefficients of 0.8790 and 0.8680 at Day 9; *ppIA* and *TPB* were ranked as the two most stable genes with Cq correlation coefficients of 0.8740 and 0.8090 at Day 12 (Table 2).

Delta Cq analysis [21] is similar to the geNorm program in that pairs of genes are compared using Cq differences. This statistical algorithm ranks the stability of reference genes by comparing the Cq value differences between two reference genes from different samples. Results are shown in Table 2. In brief, the best reference genes were *ARBP* and *TPB* with mean standard deviations of 0.34 and 0.40, respectively, at Day 9, and *ACTB* and *YWHAZ* with mean standard deviations of 0.42 and 0.45, respectively, at Day 12.

As the four statistical algorithms produced different results, the RankAggreg package was applied to determine a consensus ranking by comparing the rankings produced by geNorm, NormFinder, BestKeeper and the comparative delta-*C*t method (Table 3). *ARBP* and *ACTB* were determined to be the most suitable reference genes at Day9and *GAPDH* and *YWHAZ* at Day 12. Furthermore, the RankAggreg output is comprised of two time points and determines *ACTB* to be the most suitable reference genes for the two time point.

**Table 3:** Ranking of candidate reference genes by stability values.

**Day 9**					
**Ranking**	**geNorm**	**NormFinder**	**BestKeeper**	**Delta-*C*t**	**Consensus**
1	*ARBP* and *HPRT*	*ARBP*	*ppIA*	*ARBP*	*ARBP*
2	-	*ACTB*	*ARBP*	*TPB*	*ACTB*
3	*YWHAZ*	*TPB*	*GAPDH*	*GAPDH*	*TPB*
4	*TPB*	*YWHAZ*	*YWHAZ*	*HPRT*	*YWHAZ*
5	*Rpl13A*	*HPRT*	*HPRT*	*ACTB*	*HPRT*
6	*ACTB*	*Rpl13A*	*ACTB*	*Rpl13A*	*GAPDH*
7	*GAPDH*	*GAPDH*	*Rpl13A*	*ppIA*	*RpI13A*
8	*ppIA*	*ppIA*	*TPB*	*YWHAZ*	*ppIA*
**Day 12**					
**Ranking**	**geNorm**	**NormFinder**	**BestKeeper**	**Delta-*C*t**	**Consensus**
1	*GAPDH* and *YWHAZ*	*ACTB*	*ppIA*	*ACTB*	*ACTB*
2	-	*YWHAZ*	*TPB*	*YWHAZ*	*YWHAZ*
3	*ACTB*	*ppIA*	*ACTB*	*TPB*	*GAPDH*
4	*ARBP*	*TPB*	*ARBP*	*GAPDH*	*TPB*
5	*ppIA*	*HPRT*	*HPRT*	*ARBP*	*ARBP*
6	*TPB*	*GAPDH*	*YWHAZ*	*ppIA*	*ppIA*
7	*HPRT*	*ARBP*	*GAPDH*	*HPRT*	*HPRT*
8	*Rpl13A*	*Rpl13A*	*Rpl13A*	*Rpl13A*	*RpI13A*
**Two Time Points**					
**Ranking**	**Day 9**		**Day 12**		**Consensus**
1	*ARBP*		*ACTB*		*ACTB*
2	*ACTB*		*YWHAZ*		*ARBP*
3	*TPB*		*GAPDH*		*TPB*
4	*YWHAZ*		*TPB*		*YWHAZ*
5	*HPRT*		*ARBP*		*GAPDH*
6	*GAPDH*		*ppIA*		*HPRT*
7	*RpI13A*		*HPRT*		*ppIA*
8	*ppIA*		*RpI13A*		*RpI13A*

### 2.4. Assessment of the Validity

We selected two targets genes—brain-derived neurotrophic factor (*BDNF*) and aldolase C (*AldoC*)—to evaluate the impact of reference gene by determining the relative gene expression during 12 days. *BDNF* regulates neuronal development, survival and death [22] and plays a fundamental role in synaptic morphogenesis and function in brain regions relevant to learning and memory, such as the hippocampus [23]. *BDNF* also regulates neuronal plasticity and increases synaptic strength by inducing specific protein synthesis in dendrites [24]. *AldoC* [25] catalyzes the cleavage of fructose 1,6-bisphosphate into d-glyceraldehyde phosphate and dihydroacetone phosphate. The relative expressions of the two target genes were evaluated using the most stable gene combination (*ACTB* and *YWHAZ*) *vs.* the least stable gene *RpI13A*. When using *ACTB* and *YWHAZ* as reference genes, the relative expressions of *BDNF* and *AldoC* were found to be down-regulated (Table 4), which is similar to previous reports [18,26]. In contrast, when choosing *RpI13A* as the reference gene, the relative expressions of the two target genes were to be found up-regulated.

**Table 4:** Relative gene expression ratios of *BDNF* and *AldoC*.

Reference Genes at Day 12	*BDNF*	*AldoC*
	(*p*-value, *n*)	(*p*-value, *n*)
*ACTB* and *YWHAZ*	0.74	0.086
	(0.002, *n* = 12)	(0.612, *n* = 12)
*RpI13A*	1.37	1.95
	(0.075, *n* = 12)	(0.873, *n* = 12)

## 3. Discussion

A systematic approach has been reported by Swedish researchers, where geNorm was used to identify the most stable reference genes in BDV-infected cat brains [27]; our study, however, is the first comparison of different normalization approaches using RT-qPCR data in virus-infected primary rat cortical neurons. In order to validate the appropriate reference genes in these cells infected with a human BDV strain, we analyzed the expressions of 10 commonly-used candidate reference genes (*HPRT*, *YWHAZ*, *TPB*, *Rpl13A*, *GAPDH*, *ACTB*, *PPIA*, *ARBP*, *18sRNA* and *B2M*). During the RT-qPCR validation stage, we found that *B2M* and *18s rRNA* were not suitable as candidate reference genes. *B2M* gene expression has previously been found to be up-regulated both in brains of newborn BDV-infected rats, as well as in BDV-infected hippocampal slice cultures [28]. Then, the expression stability of the remaining eight candidate reference genes was analyzed by four algorithms. As the different normalization approaches did not provide the same results, the RankAggreg package was used to obtain a consensus ranking order of the reference genes. Since BDV infection was not detectable at Day 3 and less than 5% of neurons were positive for BDV-N at Day 6, we choose Days 9 and 12 for selecting reference genes. At Day 9, *ARBP* was ranked as the best by three algorithms, except BestKeeper, which ranked ARBP as the second-most stable gene. Interestingly, although *GAPDH* is a commonly-used reference gene in qPCR analysis [29,30], in our studies, this gene turned out to be not a good stable gene by geNorm and NormFinder. At Day 12, *ACTB* was chosen to be the most stable gene by NormFinder and BestKeeper and was ranked as the best consensus result.

The four software programs used here to determine stability in gene expression produced different results due to their different statistical outputs, namely *M*-values obtained from geNorm, variability measurements from NormFinder, coefficients of correlation from BestKeeperand mean standard deviations from the delta-*C*t method. Thus, the RankAggreg software package was used to combine these four algorithms to establish a consensus ranking among the genes. Specifically, the brute force method (*i.e*., the BruteAggreg function) was used to enumerate all possible candidate lists and then to select the one with the minimum Spearman foot rule distance [31]. This method produced the most stable and least stable reference genes for each time point. Across the two time points, RankAggreg did not output the same reference genes (Table 3). The structure and physiological function of neurons vary over time. For neurons to be connected, they need to develop an intricate structure based on neuronal processes, known as dendrites and axons [32]. Neuronal structure allows interconnections among cells and, therefore, the transmission of information. Some physiological functions and physicochemical properties of neuron may change. For instance, the electrophoretic pattern of the large microtubule-associated protein, MAP2, changes during rat brain development [33]. Besides, the process of neuron growth is influenced by the interactions of the cell with its environment. BDV replicating and spreading is different in primary neurons over time. Viral dissemination occurs after primary infection. BDV infection was first detectable on Days 4 to 6, when 5% of all neurons were positive for BDV-N. Between Day 6 and Day 10, BDV spread rapidly, and by Day 12, 100% of the neurons were infected. The distribution pattern of BDV-positive neurons strongly suggested that cell-cell contact was required for virus spread [34]. In fact, it has been reported that the expression of some reference genes may not be consistent completely over time under some conditions [14,35,36].

There are several limitations of this study to be noted here. First, this study had a limited sample size (*n* = 12 for each time point). Second, we only analyzed ten commonly-used candidate reference genes here, so better combinations of reference genes may exist. It remains to be determined whether other traditional reference genes may be more suitable as reference genes for BDV research. Third, another limitation concerns that only one type of cells was investigated for the suitability of reference genes for qRT-PCR. We did not include the knowledge on the analysis of RNA extracted from rat and mouse brains infected with BDV. However, studying and standardizing parameters in neurons (here, represented by neural cultures *in vitro*), which are the major target cells for Borna disease virus in nature, provides a further step in unraveling the complex pathogenesis of this virus disease [37].

## 4. Experimental Section

### 4.1. Primary Culture of Neurons and Viral Infections

To isolate neonatal cerebral cortices form Sprague-Dawley rats (postnatal Day 1), we used cells extracted from the brain of 16 SD rats for all 24 wells (6 infected *vs*. 6 uninfected wells at each time point, Days 9 and 12), then mixed the cells and evenly distributed them into wells. In short, the brains were taken out and submerged into ice-cold Ca^2+/^Mg^2+^-free Hanks’ salt solution (HSS), pH 7.5. After removal of the meninges, the cerebral cortical regions were dissected and dissociated by mild trypsinization (0.25% trypsin, Gibco, Shanghai, China) and DNase I (100 U/mL, Gibco) for 25 min. The cell fraction was suspended in Dulbecco’s Modified Eagle Medium (DMEM, Gibco) supplemented with 10% fetal calf serum (Gibco), 10% horse serum (Gibco), 1% glutamine (HyClone, Shanghai, China), 0.1% penicillin (10 U/mL, HyClone) and 0.1% streptomycin (10 μg/mL, HyClone). Cells were seeded at a density of 5.0 × 10^5^ cells/well on poly-l-lysine-coated6-well plates (Sigma, Shanghai, China). After 4–6 h later, the culture medium was replaced with neurobasal medium (Gibco), including 2% B-27 (Gibco) for 12 h. After removal of this medium, the cells were infected for 2 h with a multiplicity of infection (MOI) of 0.02 focus-forming units. BDV infection was performed by adding cell-released virus (CRV) to the culture medium. CRV stocks were prepared as previously described [34]. The BDV Hu-H1 strain (in oligodendroglia cell line) were kindly supplied by Hanns Ludwig (Free University of Berlin, Berlin, Germany) and is one out of the first 3 human strains derived from mentally-diseased patients [38]. These strains have partially been characterized by sequencing [39]. Then, excess virus was removed by washing with 5 mL phosphate buffer saline (HyClone) before bathing the neurons in neurobasal medium. The cells were then incubated under the same conditions for the remainder of the study. The purity of neurons was assessed by staining with the neuron-specific marker MAP-2. BDV infection of neurons was verified by immunofluorescence for each experiment.

### 4.2. Immunofluorescence

Standard tests were performed as described previously [40,41]. Briefly, both BDV-infected and control neurons in 6-well plates were incubated for 30 min at room temperature with 4% paraformaldehyde followed by permeabilization for 5 min in 0.25% Triton X-100. Then, both neuron groups were rinsed with phosphate buffer saline (PBS) 3 times and blocked with 5% bovine serum albumin (BSA) for 30 min, followed by incubation for 1 h at room temperature with the neuron-specific marker MAP-2 and a BDV-specific anti-P40 monoclonal antibody [42]. After several washes with PBS, a 1-h incubation with secondary antibodies at room temperature followed. After 3 PBS washes again, the cells were assayed using an inverted fluorescence microscope (Nikon, Tokyo, Japan).

### 4.3. RNA Isolation and Reverse Transcription

RNA was extracted from the cells using the TRIzol^®^ Reagent (Life technologies, Gaithersburg, MD, USA) according to the manufacturer’s instructions. The samples were dissolved in 20 μL DNase/RNase-free H_2_O and stored at −80 °C until use. The concentration of total RNA was determined by measuring the optical density (OD) at 260 nm, and the purity was based on the 260 nm/280 nm ratio with expected values between 1.8 and 2.0. Total RNA integrity was assessed by electrophoresis on 2% (*w*/*v*) agarose gels, as indicated in the MIQE guidelines [43]. For cDNA synthesis, first strand cDNA was prepared from 300 ng total RNA in a total volume of 20 µL using PrimeScript™ RT reagent Kit (TaKaRa, Shanghai, China). According to the manufacturer’s instructions, the reaction mixture consisted of 4 µL 5× PrimeScript Buffer, 1 µL PrimeScript RT Enzyme Mix, 1 µL OligodT Primer, and 300 ng RNA template in a total volume of 20 µL. Reverse transcription was performed in a Gene Amp PCR System 9700 (Applied Biosystems, Foster City, CA, USA) at 37 °C for 15 min and 85 °C for 5 s. The products were stored immediately at −20 °C for later use.

### 4.4. Quantitative Real-Time PCR

The qPCR reactions were performed with the ABI Prism7900 system (Applied Biosystems, Foster City, CA, USA) using the SYBR^®^ Premix Ex TaqTM II (TliRNaseH Plus, Exiqon, Vedbæk, Denmark) according to the manufacturer’s protocol and the MIQE guidelines [31]. The reaction mixture consisted of 10 µL SYBR^®^ Primer Ex TaqII, 0.5 µL PCR Forward Primer (10 µM), 0.5 µL PCR Reverse Primer (10 µM), 2 µL cDNA template and 7 µL sterile distilled water. The control cDNA synthesis reaction, without reverse transcriptase enzyme, was performed to test that the extracted RNA was not contaminated with genomic DNA. The quantitative real-time PCR reaction started at 95 °C for 30 s, followed by 40 cycles at 95 °C for 5 s and 60 s at 60 °C After that, a melting curve was performed at the end of the PCR run over a range of 55–99 °C, increasing the temperature stepwise by 0.5 °C every 2 s. A dilution series was created with random cDNA from the sample group to construct relative standard curves for each internal control gene. All samples (each group *n* = 6, total *n* = 24) were diluted 10 times and measured in triplicate.

### 4.5. Data Analysis

Statistical analysis was carried out with SPSS 21.0 statistical software (SPSS Inc., Chicago, IL, USA). Statistical significance was determined using the Student’s *t*-test and the Wilcoxon–Mann–Whitney test. *p* < 0.05 was considered statistically significant. To select a suitable reference gene, the stability of mRNA expression of each reference gene was statistically analyzed with 4 software packages: geNorm, NormFinder, BestKeeper and the comparative delta-*C*t method. Except BestKeeper and the comparative delta-*C*t method, Cq values were transformed into RQ values according to the delta-*C*t formula: RQ = E**^−^**^deltaCq^ = E^(meanCq − sampleCq)^ as input data in geNorm and NormFinder. Applying the RankAggreg package of the R project, the stability measurements produced by the above four methods were combined to establish a consensus rank of the housekeeping genes.

## 5. Conclusions

This is the first study to comparatively evaluate reference gene expression for the normalization of mRNA qPCR expression data in BDV-infected rat cortical neurons. *ARBP* was the most stable internal control gene at Day 9 and *ACTB* at Day 12. Assessment of the validity of the selected reference genes confirms the suitability of applying a combination of the two most stable references genes at each time point. This study can contribute to improving BDV research by providing a means by which to obtain more reliable and accurate gene expression measurements.
